# Asymptomatic *Plasmodium vivax* malaria in the Brazilian Amazon: Submicroscopic parasitemic blood infects *Nyssorhynchus darlingi*

**DOI:** 10.1371/journal.pntd.0009077

**Published:** 2021-10-29

**Authors:** Gregório Guilherme Almeida, Pedro Augusto Carvalho Costa, Maísa da Silva Araujo, Gabriela Ribeiro Gomes, Alex Fiorini Carvalho, Maria Marta Figueiredo, Dhelio Batista Pereira, Mauro Shugiro Tada, Jansen Fernandes Medeiros, Irene da Silva Soares, Luzia Helena Carvalho, Flora Satiko Kano, Marcia Caldas de Castro, Joseph Michael Vinetz, Douglas Taylor Golenbock, Lis Ribeiro do Valle Antonelli, Ricardo Tostes Gazzinelli

**Affiliations:** 1 Laboratório de Biologia e Imunologia de Doenças Infecciosas e Parasitárias, Instituto René Rachou, Fundação Oswaldo Cruz, Belo Horizonte, Minas Gerais, Brazil; 2 Laboratório de Imunopatologia, Instituto René Rachou, Fundação Oswaldo Cruz, Belo Horizonte, Minas Gerais, Brazil; 3 Laboratório de Entomologia, Fiocruz Rondônia, Porto Velho, Rondônia, Brazil; 4 CT vacinas, Universidade Federal de Minas Gerais e Instituto René Rachou, Fundação Oswaldo Cruz, Belo Horizonte, Minas Gerais, Brazil; 5 Ambulatório de Malária, Centro de Pesquisa em Medicina Tropical, Porto Velho, Rondônia, Brazil; 6 Departamento de Microbiologia, Imunologia e Parasitologia, Universidade Federal de São Paulo, São Paulo, São Paulo, Brazil; 7 Laboratório de Biologia Molecular e Imunologia da Malária, Instituto René Rachou, Fundação Oswaldo Cruz, Belo Horizonte, Minas Gerais, Brazil; 8 Department of Global Health and Population, Harvard T.H. Chan School of Public Health, Boston, Massachusetts, United States of America; 9 Section of Infectious Diseases, Department of Internal Medicine, Yale School of Medicine, New Haven, Connecticut, United States of America; 10 Department of Medicine, University of Massachusetts Medical School, Worcester, Massachusetts, United States of America; Johns Hopkins Bloomberg School of Public Health, UNITED STATES

## Abstract

Individuals with asymptomatic infection due to *Plasmodium vivax* are posited to be important reservoirs of malaria transmission in endemic regions. Here we studied a cohort of *P*. *vivax* malaria patients in a suburban area in the Brazilian Amazon. Overall 1,120 individuals were screened for *P*. *vivax* infection and 108 (9.6%) had parasitemia detected by qPCR but not by microscopy. Asymptomatic individuals had higher levels of antibodies against *P*. *vivax* and similar hematological and biochemical parameters compared to uninfected controls. Blood from asymptomatic individuals with very low parasitemia transmitted *P*. *vivax* to the main local vector, *Nyssorhynchus darlingi*. Lower mosquito infectivity rates were observed when blood from asymptomatic individuals was used in the membrane feeding assay. While blood from symptomatic patients infected 43.4% (199/458) of the mosquitoes, blood from asymptomatic infected 2.5% (43/1,719). However, several asymptomatic individuals maintained parasitemia for several weeks indicating their potential role as an infectious reservoir. These results suggest that asymptomatic individuals are an important source of malaria parasites and Science and Technology for Vaccines granted by Conselho Nacional de may contribute to the transmission of *P*. *vivax* in low-endemicity areas of malaria.

## Author summary

Malaria still poses as one of the most important parasitic diseases in the world. The advance of molecular diagnosis brought to light the existence of asymptomatic infections, which may represent most of the infections in some areas. Importantly, the role of asymptomatic carriers in the natural history of malaria is not completely understood. Herein we describe the general characteristics of asymptomatic individuals infected with *Plasmodium vivax*, and provide evidence of their potential as parasitic reservoirs, even when molecular methods fail to detect the infection. Our findings reinforce the need for better diagnostic tests and open a new window of complexity to be considered in control programs.

## Introduction

*Plasmodium vivax* is spread worldwide and although it is frequently considered to be low pathogenic, it is an important cause of morbidity and mortality in endemic areas in central and south America, and in regions of Asia and Oceania [1,2]. The annual incidence of vivax malaria has reduced since the last century in Brazil, but to achieve elimination new strategies are needed to optimize diagnostic and early treatment, and to block transmission from humans to mosquitoes [3]. Control of *P*. *vivax* is challenging due to the unique biology of this species, including the early emergence of infectious gametocytes within 3 days after the detection of asexual forms, the development of hypnozoites in the liver, a potential source of later relapses, and the preferential infection of reticulocytes, which represents 0.5–2.5% of the red blood cells, which limits the parasite density [4,5].

Patients with symptomatic malaria actively seek diagnosis and treatment. In contrast, asymptomatic (ASY) cases of *P*. *vivax* infection are mostly undetectable, neglected, and remain untreated. For this reason, asymptomatic malaria require active surveillance and poses as one of the most challenging obstacles for the control of *Plasmodium* infections worldwide [6,7]. Evidence has shown that asymptomatic individuals commonly have very low parasite density, usually undetectable by field tests, such as microscopy or rapid diagnostic tests (RDTs) [8]. Although the role of these submicroscopic infections in the maintenance of endemicity is not well understood, it is generally thought that they may serve as parasitic reservoirs sustaining endemicity and causing new outbreaks [9–11].

In the Brazilian Amazon, the incidence of malaria dropped from near 600,000 cases/year in the late 1990s to near 144,000 in 2014 [3], mostly due to the implementation of the control measures established by the National Malaria Control Programme. Despite these recent declines, malaria is still hypoendemic in the Amazon and asymptomatic cases are thought to be one of the obstacles to the elimination of *P*. *vivax* transmission in the Amazon basin in Brazil [12]. Hence, considering the global effort to eliminate malaria, it is crucial to characterize the asymptomatic population and to better understand if and how submicroscopic infections impact the transmission and impair the control of the disease. Herein we verified the frequency and the spatial distribution of asymptomatic infection in a low endemic setting of malaria caused by *P*. *vivax* in the Brazilian Amazon and explore their characteristics, which will be relevant for future studies in this population.

## Methods

### Ethics statement

This study was performed under protocols reviewed and approved by the Ethical Committees on Human Experimentation from Instituto René Rachou, Fiocruz, and National Ethical Council (CAAE: 59902816.7.0000.5091). All participants were informed about the objectives and procedures of the study, with voluntary participation through written informed consent.

### Surveys and inclusions criteria

#### Screening for asymptomatic infection

Candeias do Jamari (8°47’41.6"S 63°42’10.9"W) is a municipality localized in the south-western Amazon region in Brazil, where malaria is endemic and the annual parasite index usually exceeds 50 cases per 1,000 inhabitants. Between 2018 and 2019, 2,394 cases of *P*. *vivax* and 287 cases of *P*. *falciparum* were notified in this town [13].

From December 2018 to October 2019, four cross-sectional surveys were done in 3 areas from this municipality to identify asymptomatic infections. Candeias do Jamari has two main seasons: a wet season between October and April and a dry season between May to September. Transmission of *P*. *vivax* occurs during the entire year, although peaks of transmission occur during the dry season. Areas 1 and 2 had the most reported symptomatic cases in the past years, while area 3 has reported fewer cases of symptomatic malaria [14]. Screenings were done in a household-based sampling comprising adult individuals (18 years or older) manifesting interest in being enrolled in the study, reporting or having no symptomatic malaria in the last two months and at the time of enrollment. Exclusion criteria included: pregnancy, breastfeeding, and current diagnostic of chronic inflammatory or infectious diseases.

After informed consent, 200μL of blood were collected by finger puncture and Giemsa-stained thick blood smears were performed. Blood was centrifuged, and DNA was extracted from compacted cells using a commercial kit (QIAmp DNA blood mini kit, Qiagen). Extracted DNA was resuspended in 200μL of Elution Buffer to reproduce the original concentration of parasites/μL. Samples were tested at least in triplicate by qPCR and individuals with positive results for *P*. *vivax* in at least one replicate were included in the Baseline study. No template controls (NTC) were included in all reactions to ensure the reliability of the results.

#### Baseline and longitudinal arm

Four to seven days after the first positive qPCR for *P*. *vivax* at Screening, 47 from 108 ASY donated a new blood sample, were interviewed, and clinically examined by a physician. This date of clinical examination was considered as the Baseline in our analysis. Thirty-eight symptomatic (SY) individuals with positive thick blood smears for *P*. *vivax* performed at the Centro de Pesquisas em Medicina Tropical (CEPEM) were also enrolled in this study. Twenty healthy individuals from Porto Velho with negative parasitemia by thick blood smear and PCR, and reporting no malaria symptoms for more than two years were enrolled as controls (CTL).

Blood was collected by venipuncture, and samples were used for routine blood counting and clinical biochemistry, DNA extraction, and thick smears for parasite detection. Interviews were done using a web-based tool for research data curatorship (REDCap). Heparinized blood samples from 32 ASY (17 from the Baseline and 15 of the last time point of the longitudinal arm, described below) and 9 SY were used in the membrane feeding assay. In this case, volunteers were included according to the availability of mosquitoes to perform the experiment. Treatment was offered to all ASY and those who chose to be treated received 3 days of chloroquine (600 mg on day 1, and 450 mg on days 2 and 3) and primaquine 7 days (total dose 3–4.2 mg/kg).

**Fig 1:**
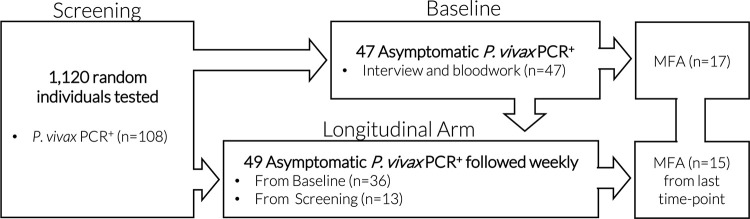
Study design. From 1,120 randomly tested individuals 108 tested positive in qPCR for *P*. *vivax* from which interviews and blood sampling were done in 47 at the Baseline. Longitudinal arm included 36 and 13 ASY individuals from Baseline and Screening, respectively. Membrane feeding assay was performed in 17 ASY from the Baseline and 15 from the last timepoint of the longitudinal arm.

Forty-nine individuals were followed weekly for six weeks to assess the development of symptoms and for parasite detection (longitudinal arm). Among those, 36 subjects were from the Baseline, and additional 13 subjects were from Screening. The study design is depicted in Fig 1.

### Molecular diagnoses of *Plasmodium vivax* infections

A qPCR addressed to a sub-telomeric non-ribosomal multicopy target (Pvr47) was done as described by Amaral *et al*. [15], with modifications. Briefly, 2 μL of extracted DNA was mixed with 0.5 μM of primers (Pvr47-Forward 5’-TCCGCAGCTCACAAATGTTC3-3’, Pvr47-Reverse 5’-ACATGGGGATTCTAAGCCAATTTA-3’), 0.25 μM of probe (fam-5’-TCCGCGAGGGCTGCAA-3’-MGB), 5 μL master mix (CTVacinas, Brazil) and 1 μL of PCR-grade water. Reactions were done in an Applied Biosystems 7500 Real-Time PCR System with the following steps: 2 minutes of initial denaturation at 95°C followed by 45 cycles of denaturation (95°C/3s), annealing (54°C/30s) and extension (60°C/30s). Positive, negative, and blank controls were added to each plate. The same positive control was added to each plate to ensure the parasite quantification comparability inter-assay. A standard curve was built using a TOPO-2.1 plasmid with the amplified fragment inserted to calculate the number of copies of the target in a sample.

The PCR for *P*. *falciparum* was performed only in samples that were PCR positive for *P*. *vivax* to avoid the inclusion of patients co-infected with both species [15]. When blood samples were PCR negative for *P*. *vivax*, but infected mosquitoes during membrane feeding assay (MFA), we also employed a nested PCR to confirm the result [16]. Briefly, 2 μL DNA samples were mixed with 0.4 μM each SSUrRNA universal primers (P1:5’- ACGATCAGATACCGTCGTAATCTT-3’ and P2: 5’-GAACCCAAAGACTTTGATTTCTCAT- 3’), 125 μM each of dNTPs, 2.5 mM MgCl_2_, 1x Taq buffer (Promega), and 0.75 units of Taq polymerase (Promega), in a 20 μL final volume. Amplification was done as follows: 92°C/2m, followed by 35 cycles of 92°C/30s and 60°C/90s and a final extension of 60°C/5m. The product was diluted 1:50 in TE buffer (Qiagen) and 2 μL were applied in 8 μL mix containing 1 μM of primer P1 and a *P*. *vivax* primer (Vi, 5’-CAATCTAAGAATAAACTCCGAAGAGAAA-3’), 312.5 μM of each dNTP, 1x Taq buffer (Promega), 1.5 mM MgCl_2_, and 0.75 units of Taq polymerase (Promega). Amplification was as follows: 92°C/2m, followed by 18 cycles of 92°C/30s and 60°C/60s and a final extension of 60°C/5m. Products were electrophoresed and seen as ~110 bp bands in a 2% Agarose gel in a UV transilluminator.

### Enzyme-linked immunosorbent assay

Plasma from 47 ASY, 38 SY, and 20 healthy donors that reported never had malaria before was used to assess the levels of IgG against the *P*. *vivax* blood-stage antigens *Pv*MSP-1_19_ [17], *Pv*AMA-1_66_ [18] and *Pv*DBP-II_Brz-2_[19] by ELISA as described [20]. The results were expressed as reactivity index (RI = the ratio between the OD 450nm values obtained from the sample and the cut-off value). Cut-off value was set at 3 standard deviations above the mean OD 450nm of plasma from 34 individuals from the south-east of Brazil, a non-endemic area for malaria, and 12 individuals who reported never having had malaria before, from Porto Velho, the capital of Rondônia located 20km from Candeias do Jamari. Values of RI higher than 1.1 were considered positive.

### Human-to-Mosquito transmission experiments

*Nyssorhynchus darlingi* mosquitoes from an established colony (F8-F18 generations) were reared under standard laboratory conditions as described [21]. *N*. *darlingi* is the main vector of *P*. *vivax* in the area we studied. Mosquitoes were maintained on cotton soaked with 15% honey solution *ad libitum* one day before and after the infection blood meal. Heparinized blood was kept at 37°C, for approximately 10 minutes, until the membrane feeding assay. Blood sample (2mL) was loaded into a glass feeder closed with parafilm membrane and maintained at 37°C by a connected heated water source. One mesh-covered 650 mL plastic cup containing 80 females 3- to 5- days old *N*. *darlingi* (F8 to F18 generations) that had been starved for 24 h, were placed under the feeder for 30 min to allow feeding. Fully engorged females were transferred into 20cm^3^ cages and maintained in the same conditions of insectarium for 2 weeks.

One-third to one-half of each group of mosquitoes were dissected on day 7 and the remaining mosquitoes on day 14 post-feeding. Mosquitoes were freeze-killed and their midguts were dissected out in phosphate-buffered saline and stained with 0.2% w/v mercurochrome (in ddH_2_O) for 5 min. The percentage of mosquitoes infected and the number of oocysts/mosquito were determined by examination at 40x objective on a light microscope.

### Statistical analysis

Differences between proportions were calculated by Fisher’s exact test. Odds ratios and confidence intervals (95%) were calculated by the Baptista-Pike method. Maps were edited using the software Q-GIS 3.12 and employing the SIRGAS 2000 UTM 22s coordinate system. Base-layer was from the ArcGIS (source: Esri, Maxar, GeoEye, Earthstar Geographics, CNES/Airbus DS, USDA, USGS, AeroGRID, IGN, and the GIS User Community. Available on https://arcg.is/1H5GjP). Variables were tested for Gaussian distribution using the Shapiro-Wilk test and variables were tested according to their distribution. Mann-Whitney U test and t-student test were used for comparisons between two groups. ANOVA or Kruskal-Wallis tests were used for multiple comparisons. The Spearman’s rank correlation coefficient was used to test correlations. These analyses were performed using GraphPad Prism 8 (GraphPad Software, San Diego, CA, USA). Longitudinal spot-matrix were done using R and ggplot2 package. The statistical significance threshold was P < 0.05, with 95% confidence intervals for all hypotheses tested.

## Results

### Occurrence of asymptomatic cases of *P*. *vivax* infection are associated with the distribution of symptomatic malaria

An overall 1,120 individuals were tested for *P*. *vivax* infection by qPCR during the 4 Screenings from which a total of 108 (9.6%) were PCR positive (Table 1). All ASY were negative in blood thick smear. The male/female ratio was 0.93 (540/580). There was a higher prevalence of ASY infection among males in December compared to females (OR 3.244, CI: 1.552–6.604, p = 0.002, Table 1). The prevalence of asymptomatic infection was higher in females in September when compared to December in the same area (OR 2.218, CI: 1.027–4.568, p = 0.046, Table 1).

**Table 1:** General data on Screening of asymptomatic individuals.

Screening	Sex	#Tested	% Positive	Overall prevalence	OR(CI)*	P-value*
**Area 1** **December/2018 (wet season)**	F	156	6.4%^a,b^	12.3%^c^	7.020 (2.615–18.60)	<0.0001
	M	154	18.2%^a^			
**Area 2** **January/2019** **(wet season)**	F	132	8.3%	7.7%^d^	4.206 (1.457–11.58)	0.0051
	M	114	7.0%			
**Area 1** **September/2019** **(dry season)**	F	182	13.2%^b^	13.1%^d^	7.570 (2.904–19.90)	<0.0001
	M	177	13.0%			
**Area 3** **October/2019** **(wet season)**	F	110	1.8%	2.0%^c,d^	-	-
	M	95	2.1%			
**Total**	_	1120	9.6%	_	_	_

Same letters correspond to significant difference between proportions in Fisher’s exact test (p<0.05).

*Odds-ratio, confidence interval and respective p-value for the overall prevalence compared to area 3.

**Fig 2:**
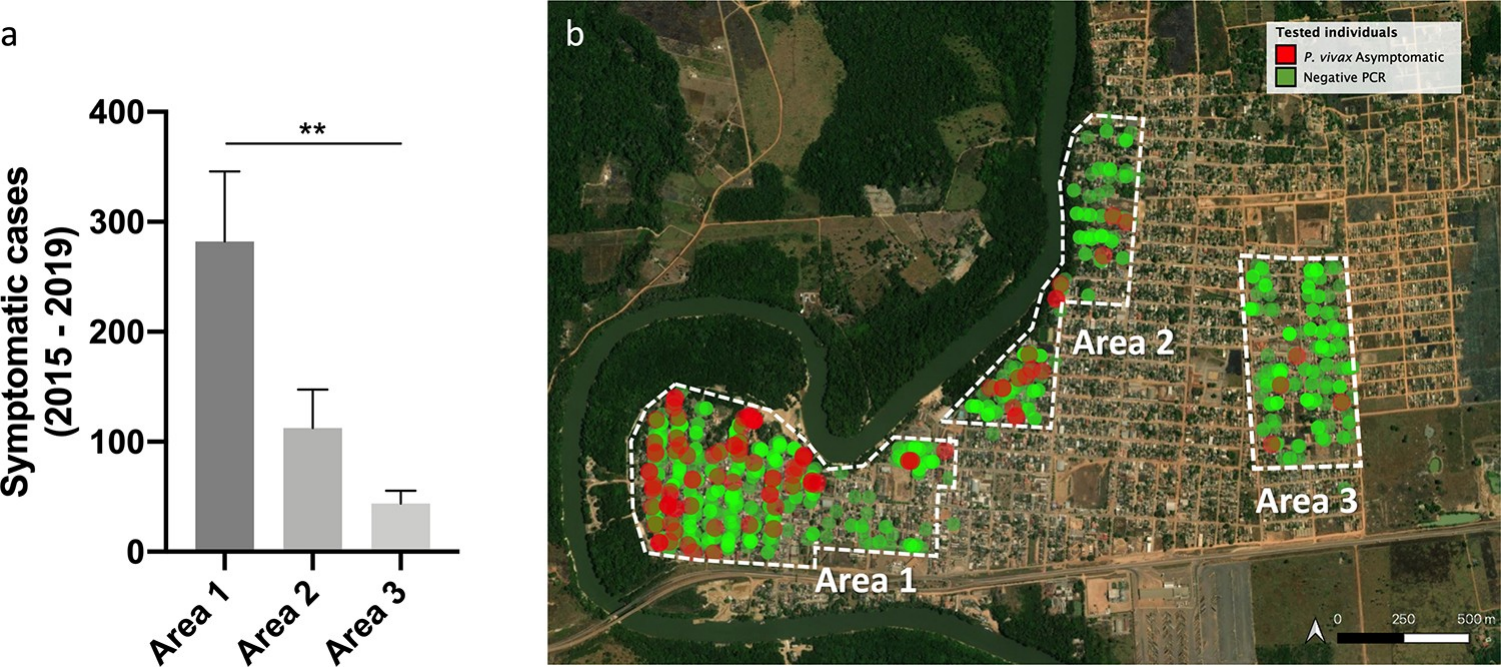
Asymptomatic individuals distribution correlate with symptomatic cases. (a) Mean and standard deviation of officially notified symptomatic cases between the studied areas from 2015 to 2019. (b) Spatial distribution of asymptomatic infections between the 3 areas studied in Candeias do Jamari, RO. Map was built using QGIS using base layers from USGS source: Esri, Maxar, GeoEye, Earthstar Geographics, CNES/Airbus DS, USDA, USGS, AeroGRID, IGN, and the GIS User Community. (Available on https://arcg.is/1H5GjP).

To test the distribution of ASY according to SY cases, the surveys conducted in December, January, and September were focused on the two areas with higher number of reported SY infection (areas 1 and 2), while in October the Screening was conducted in an area with fewer notified cases of SY malaria (area 3). We visited all houses of area 1 in both surveys, and only 64 individuals out of 669 were sampled in more than one survey. Although all houses were visited in both surveys we could not interview or sample all the residents due to the absence of people at the time of visit or sampling refusal. There was no significant difference between the percentages of ASY from areas 1 (December) and area 2 (January), but a marginally significant difference was observed between the proportions of areas 1 and 2 when September and January were compared (OR 1.8, CI 1.026–3.112, p = 0.0459). In contrast, area 3 had a lower prevalence of ASY compared to the other areas (Table 1), following the same trend found for SY cases. Interestingly, the distribution of ASY is associated with the average of annual notified cases in each studied area (Fig 2A). The spatial distribution of individuals at a household level is shown in Fig 2B.

**Fig 3:**
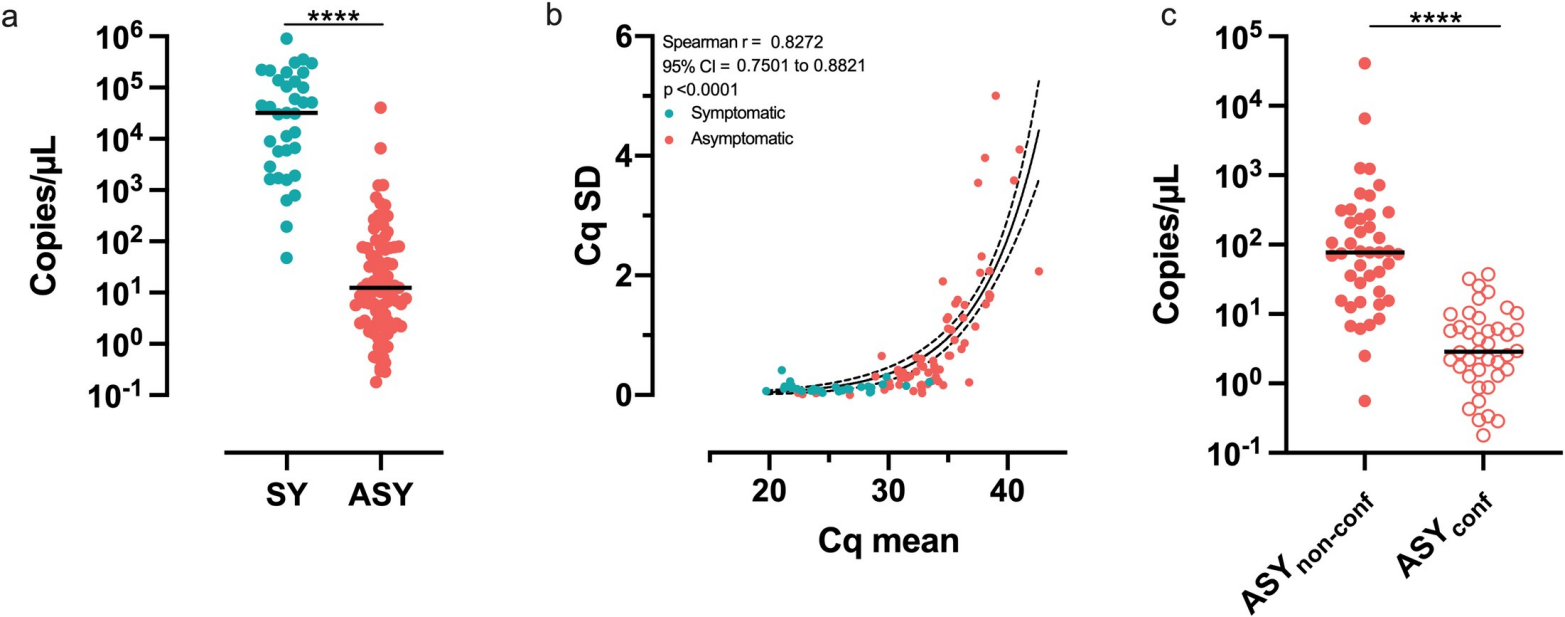
Asymptomatic infections with *Pv* are characterized by lower parasitemia compared to symptomatic patients. (a) Estimated parasitemia determined by qPCR. Asymptomatic infections are characterized by very low parasitemia. Each dot represents the average number of copies/μL of an individual in at least 3 replicates. (b) Correlation between mean Cq value and correspondent standard deviation. High Cq values correlate with higher variation in the measurement. (c) Several asymptomatic infections had conflicting PCR results (ASY_conf_); 1 or more negative results in qPCR. Asymptomatic non-conflicting (ASY_non-conf_) individuals had all qPCR replicates positive. Each dot represents the average number of copies/μL estimated from the Ct of the positive replicates of a subject, in at least 3 replicates. Bars amid dot plots represent the respective means. ****p<0.0001. t-Student test. SY–Symptomatic patients. ASY–Asymptomatic individuals.

The parasitemia in SY was higher than in ASY (Fig 3A). The median of the estimated parasite density of SY patients was 39,279 copies/μL (IQR 3,247–178,458), while for ASY individuals was 14.2 copies/μL (IQR 2.44–76.5). High variability inter- and intra-assay in the mean Cq value were observed in ASY at the higher Cqs. Moreover, no amplification was observed in one or more replicates of the same ASY sample. To test whether this finding was due to the very low parasitemia we evaluated the correlation of each mean value with the respective standard deviation of the Cq. There was a positive correlation between the standard deviation of the replicates and the cycle threshold, indicating that the lower the number of copies in the sample, the higher the variability between replicates (Fig 3B). Estimated parasitemia of ASY with conflicting results (ASY_conf_) was lower than the ASY with non-conflicting results (ASY_non-conf_) whose all replicates had detected amplification (Fig 3C).

### Most asymptomatic individuals report a previous symptomatic infection

Of the 47 ASY enrolled at Baseline, most (93%, 44/47) reported a previous episode of SY malaria, with 77% (34/44) and 61% (27/44) reporting respectively more than 2 and 5 or more symptomatic episodes in their lives. The time since the last SY malaria was higher than 12 months for 75% (33/44) of participants. The average age of ASY was 40 years old, and the male/female ratio was 0.8 (21/26). The average ages for SY and CTL were 39.2 and 28.9 years old, and the male/female ratios were 2.3 and 1.1, respectively. In addition, 49% (23/47) of the ASY had become PCR negative in about one week. No ASY had fever during clinical examination, while 41% (14/34) of the SY had an axillary temperature above 37.5°C. From the 47 ASY included, 34 (72%) were re-assessed 30 to 45 days after the Baseline enrollment, and none of them reported seeking or receiving malaria treatment in the previous month.

### Hematological and biochemical parameters are not altered during asymptomatic malaria caused by *P*. *vivax*

**Fig 4:**
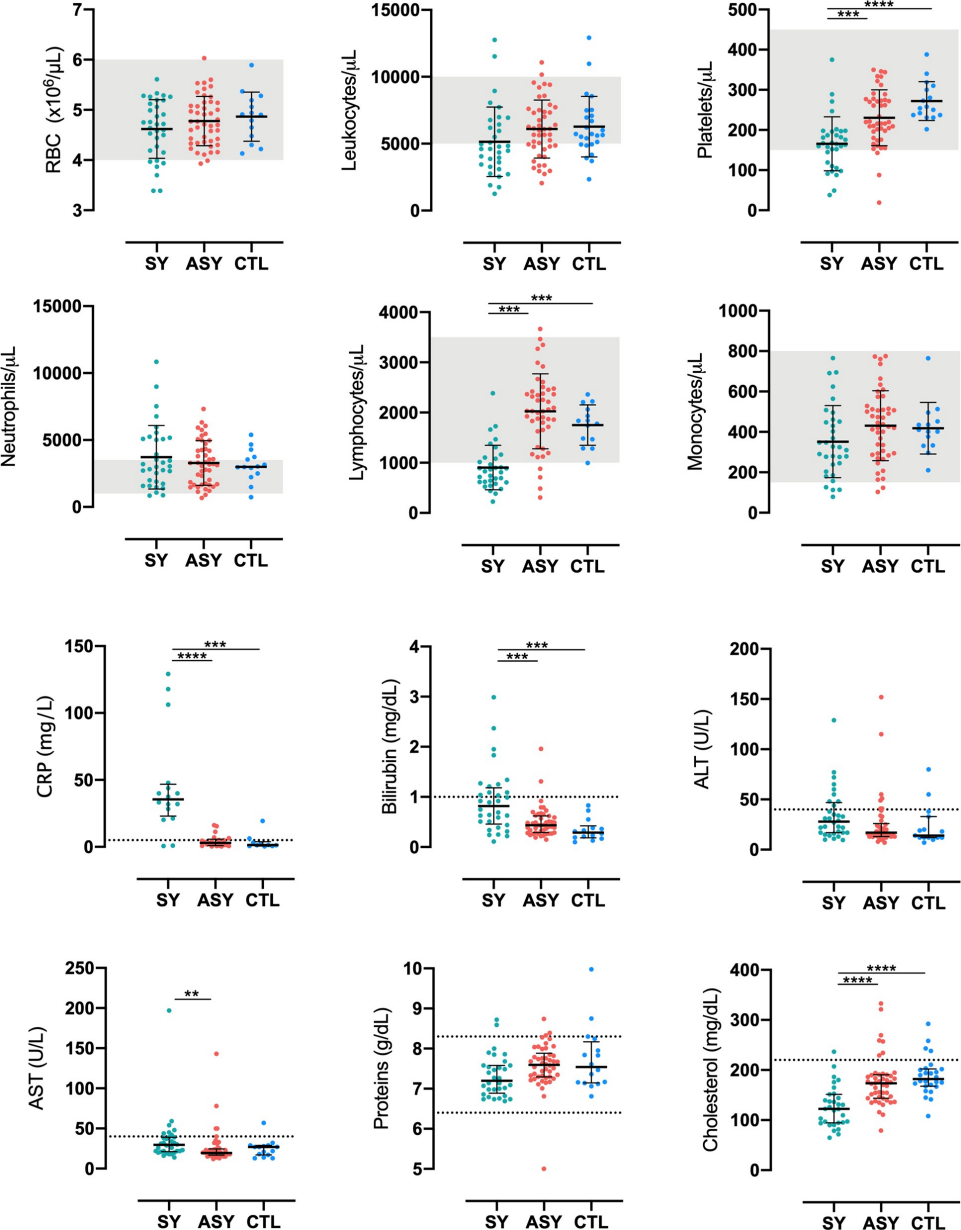
Asymptomatic individuals do not display hematological or biochemical alterations. Each dot represents individual values of each analyte by group. Gray background and dotted lines represent range of expected values in healthy individuals. Only significant differences were shown. *p<0.05. **p<0.01. ***p<0.001. ****p<0.0001. Dunn’s test. SY–symptomatic patients. AS–Asymptomatic individuals. CTL—healthy controls. CRP–C-reactive protein. ALT–alanine transferase. AST–Aspartate transferase.

No relevant routine hematological abnormality was observed in ASY. Most SY had marked lymphopenia and low counts of platelets (Fig 4). Biochemical parameters were analyzed, and levels were comparable between ASY and CTL. Symptomatic patients had higher levels of C reactive protein (CRP) and bilirubin, and lower levels of cholesterol compared to ASY and CTL (Fig 4).

### Most of the asymptomatic individuals have IgG anti-*P*. *vivax* antigens

**Fig 5:**
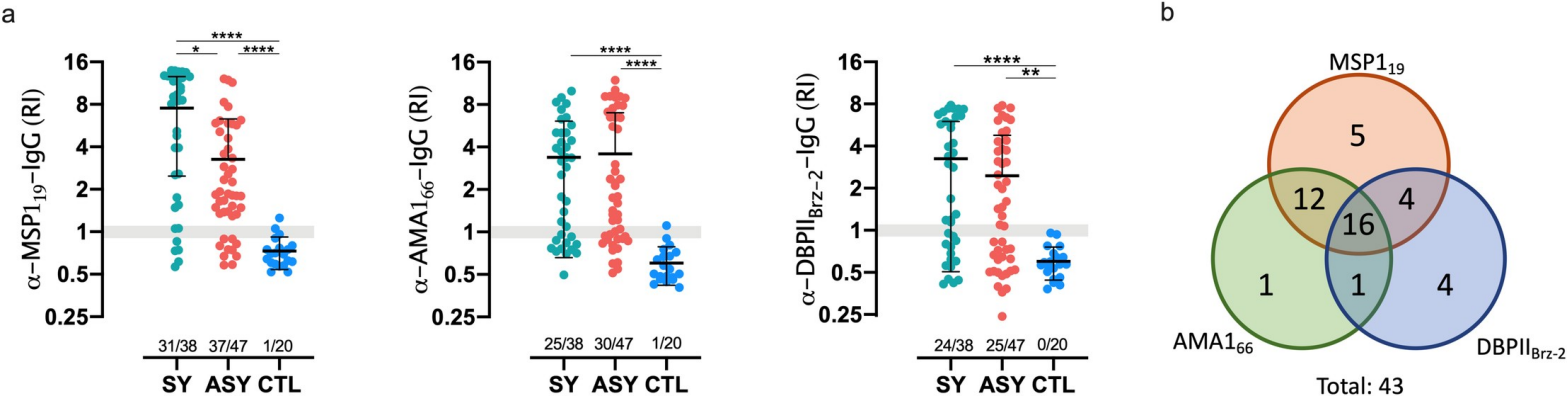
Asymptomatic individuals have higher levels of IgG against *P*. *vivax* antigens compared to healthy controls. a) Reactivity index for each individual by group for the three antigens. Each dot represents individual values of each analyte by group. Gray background represents the cut-off threshold. Data above the background is positive, and bellow, negative. Data upon the gray line is considered inconclusive. Y-axis is Log2 transformed to better show the distribution of negative individuals. Only significant differences were shown. **p<0.01. ****p<0.0001. Dunn’s test. b) Venn’s Diagram depicting the distribution of IgG positive ASY for the three antigens. RI–reactivity index. SY–symptomatic patients. ASY–Asymptomatic individuals. CTL—healthy controls.

Levels of IgG against the recombinant antigens *Pv*MSP-1_19_, *Pv*AMA1_66,_ and *Pv*DBPII_Brz-2_ were significantly higher in ASY than in CTL. Despite the levels observed in SY showed to be higher than those observed in ASY, 78% of the latter produced IgG anti-*Pv*MSP-1_19_ above the cutoff. Also, 63.8% and 53.2% of ASY displayed IgG levels against *Pv*AMA1_66_ and *Pv*DBP_II_-Brz-2 above the cutoff (Fig 5A). Interestingly, 4 individuals had no detectable IgG against the antigens, while 16 (37.2%) among IgG positive individuals had antibodies against all three antigens (Fig 5B). Noteworthy, except for *Pv*MSP-1_19_, levels of IgG were similar between ASY and SY.

### Peripheral blood infection by *P*. *vivax* can be detected for several weeks in symptomless individuals

**Fig 6:**
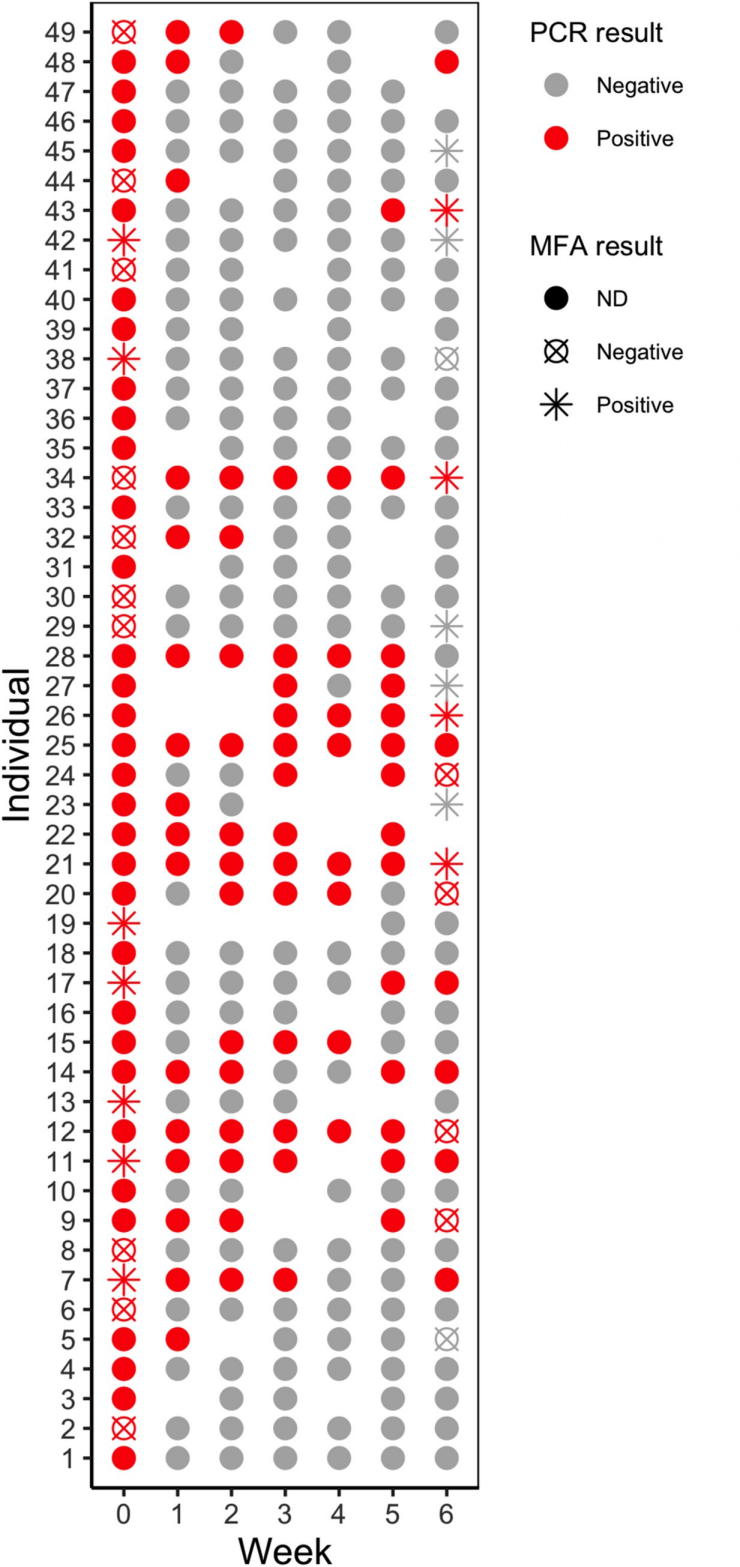
Asymptomatic individuals may stay infected for several weeks. Asymptomatic individuals were followed for weekly collection of blood from the finger. Dot matrix depicting the positive and negative PCR results for each individual over time. Missing samples are represented by missing dots.

A group of 49 ASY was followed by 6 weeks with weekly blood sampling to verify changes in parasitemia. From the 49 subjects, 27 (55%) became PCR negative as soon as one week after the sampling and 15 were negative in all samplings throughout the study. ASY that become negative in the following week, have a higher probability of bearing parasite density below the median (14.2 copies/μL) (Fisher’s exact test, p<0.0001, OR = 21.4, CI 6.03–60.82). Also, 6 subjects had at least one positive PCR after the 3^rd^ week. Twenty individuals were PCR positive in at least two consecutive weeks, and 4 were positive in all weeks tested. Parasite densities did not vary substantially in the individuals with detectable parasitemia across the weeks. While some individuals showed detectable parasitemia for several weeks, some became negative for *P*. *vivax*, showing positivity later during the longitudinal arm (Fig 6), suggesting either reinfection or oscillating parasitemia. No individual included in the longitudinal arm became symptomatic during the study.

### Blood from asymptomatic *P*. *vivax*-infected individuals infect *N*. *darlingi*

**Fig 7:**
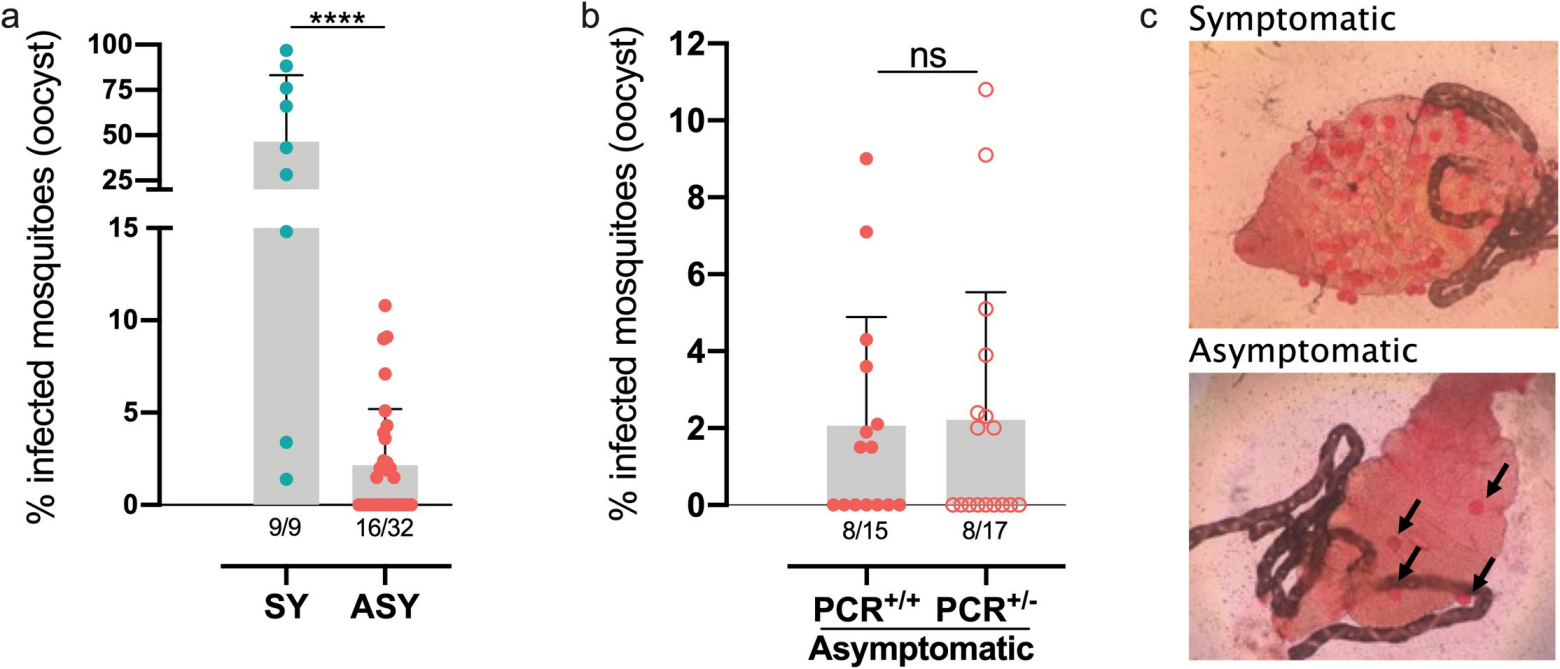
Asymptomatic individuals can infect *N*. *darlingi* even with undetectable parasitemia by qPCR. Eighty to one-hundred colony raised females of *N*. *darlingi* were fed in fresh blood collected from symptomatic patients (SY) and asymptomatic individuals (ASY), with current (PCR^+/+^) or past (PCR^+/-^) detected low parasitemia by qPCR. Each dot represents individual values of the frequency of infected mosquitoes by person. **p<0.01. ns- not significant. two-tailed Mann-Whitney U test. c) Photomicrographs showing several oocysts in midguts from mosquitoes fed in symptomatic or asymptomatic individuals (arrows).

Finally, we verified the potential of blood from ASY to infect colony-raised *N*. *darlingi*. From 2,823 engorged mosquitoes, 2,177 were dissected with a mean of 77.2% surviving until midgut dissection. Blood from all 9 SY were able to infect mosquitoes, with a rate of infection of 43.4% (199/458). The numbers of oocysts varied from 1–108 per midgut. Blood from 50% (16/32) of ASY was able to infect mosquitoes, with significantly lower infection rates compared to SY (Fig 7A). Blood from ASY infected 2.5% (43/1,719) of the mosquitoes with the numbers of oocysts varying from 1–8 per midgut (Fig 7C).

From 15 ASY with positive PCR in the same blood sample used for MFA, 8 were able to infect mosquitoes, as evidenced by the oocysts found in their midgut ranging from 1 to 7 per midgut. Interestingly, among 17 ASY who were PCR positive at Screening but became negative at the time of sampling for MFA (PCR^+/-^), blood samples from 8 ASY infected mosquitoes. The average number of infected mosquitoes was similar between both groups (Fig 7B). Most importantly, 5 out of those 8 ASY, whose blood infected mosquitoes, were PCR negative in the same samples used for MFA (Fig 6, individuals 23, 27, 29, 42 and 45 at sampling week 6). To exclude the possibility that these findings were due to DNA polymorphisms or intrinsic PCR failures, a nested-PCR addressing the 18S ribosomal gene [16] was employed in the same samples and confirmed the results.

## Discussion

Here, we found that in areas with a higher frequency of symptomatic cases, a considerable proportion of the population currently present *P*. *vivax* subpatent infections. These areas are characterized by their proximity to rivers and the riparian forest, which provides environmental conditions to maintain the life cycle of anophelines. Importantly, we found that blood from ASY with subpatent parasitemia effectively infected the vector *N*. *darling* with *P*. *vivax*.

During the rainy season (October to April), most of the riparian forest is submerged due to the flooding of Jamari river. In this period, the number of SY cases is lower when compared to the dry season (May to September) [14,22]. After the flood, infection rates rise due to the increase of the anophelines breeding habitats and numbers [23]. Historically, more SY cases are reported in September than in December [13,14]. However, our data suggest that the proportion of ASY infections does not follow the seasonality observed for SY infection, as also found by others [24].

The fact that ASY individuals have lower parasite densities compared to SY is well known [25]. The conflicting results for some of the individuals were inversely correlated with parasitemia, suggesting subsampling error or stochastic amplification (Monte-Carlo effect) [26,27]. In those cases, several replicates were necessary to conclude whether the individual was infected or not. The fact that 50% of samples tested by MFA with negative PCR were able to infect mosquitoes reinforces that these results are due to very low parasitemia and not due to sample cross-contamination [28]. The limit of detection (LOD) calculated for our qPCR was 154.2 copies/μL, detecting as low as 23.5 copies/μL by the 95%CI. Values below the LOD were extrapolated from the standard curve and therefore subjected to higher variability. Besides the intrinsic limitations of this approach, our results are compatible with those found by others for *P*. *falciparum* [29] and *P*. *vivax* [15,27,30].

Defining ASY infection is not trivial [31]. The first concern to be considered is the occurrence of pre-symptomatic patients, since the incubation period might be remarkably long [32]. To avoid this bias, we monitored ASY up to 45 days after the enrollment to ensure they remained ASY. Moreover, in the longitudinal study, no ASY received treatment or developed classical symptoms of malaria or others that made them seek medical care. No relevant alterations were found in parameters from ASY that are usually observed in SY, such as lymphopenia and thrombocytopenia, along with high levels of CRP and bilirubin and lower levels of cholesterol [33]. This finding reinforces that, along with the very low parasitemia, which might be undetectable at times, asymptomatic infections are also undetectable by routine exams or clinical evaluation.

Almost 80% of the ASY had detectable levels of IgG anti-MSP-1_19_, as previously described [34]. It has been shown that serum levels of IgG against *Pv*MSP-1_19_ slightly decline in SY 2 months after treatment [35]. Likewise, IgG against *Pv*MSP-1_19_ is also short-lived in ASY [36,37]. It is not clear if the persistence of low parasite density is enough to sustain high levels of IgG for longer periods. These results might be relevant for the development of serological tests for the diagnosis of ASY infection.

As reported by others [29,38], our data show that parasitemia can oscillate when close to the detectable limits of molecular methods. The infection status of the subjects included in this study was assessed weekly for 6 weeks. Others evaluate the infection persistance of *P*. *vivax* monthly for longer periods [39]. In both cases, no approach was used to verify if the presence of parasitemia in two different time points represents persistance or new infections. While the duration of a submicroscopic infection may be as long as five consecutive months, incubation periods of vivax malaria may be as long as one year [32,39]. The presence of the dormant phase of *P*. *vivax* (hypnozoites), may have an impact on the maintenance of low parasitemia in ASY, since a high proportion of recurrent infections evolve to ASY infections [5,40,41]. A recent study suggests that bone-marrow is an important reservoir of *P*. *vivax* in monkeys, but evidence in humans is still absent [42]. Thus, up to now, these data are not able to determine whether the current ASY infection has been maintained for a long time or if individuals have been frequently re-infected with *P*. *vivax*, sustaining the low parasitemia and boosting the antibody levels.

In this study, we used 80–100 female *N*. *darlingi* to fed on blood of each subject. Assuming an average of 2–3μL of blood ingested by mosquito [43] and with an average of 77% of engorged mosquitoes, a total of 154–231μL of blood was tested by MFA, while only an equivalent of 6–20μL of blood was tested by PCR, which might explain the discrepancies with the qPCR and the mosquito infection in individuals with negative PCR results. All SY infected mosquitoes with a load of oocyst per midgut ranging from 1–108. Another study found 50% of infectivity with a range of 1–175 oocysts per midgut using similar methodologies and the same species of Anopheline (F1)[44]. Although SY were on average more infective to mosquitoes, almost half of the ASY were infective to *N*. *darlingi* in infection rates varying from 2.2–10.8%, as previously reported for *N*. *darlingi* [11] and for other species [25,45]. Importantly, half of the individuals with a current negative PCR, but with a previous infection detected less than one week before (PCR^+/-^), were able to infect mosquitoes with the same efficiency as the currently PCR positive (PCR^+/+^) ASY. Kiattibutr *et al* [46] found a similar result in 9 samples with negative PCR results, with a rate of infection lower than the one shown here. The lack of the detection of *P*. *vivax* in some samples is likely due to the very low parasite density or the sensitivity of the molecular assays [47], but enough to allow transmission when a large number of mosquitoes are fed.

It has been shown that in *P*. *falciparum* malaria, the contribution of ASY for the transmission is higher in low transmission settings, and increases with the relative proportions of subpatent infections [29]. Another elegant study assessed the relative contribution of ASY and SY malaria for both species, *P*. *falciparum* and *P*. *vivax*, to the infectious reservoir in low-endemic settings in Ethiopia. The ASY infection by *P*. *vivax* had a greater impact on the transmission compared to SY, being responsible for the majority of the infectious reservoir [25]. Such studies were still not performed in our study area in Brazil, and thus the relative contribution of ASY *P*. *vivax* infection in our settings remains elusive. Also, it is important to notice that only adults were included in this study, and the contribution of asymptomatic malaria in children (below 18 years old) to transmission is unknown in this setting.

Very low numbers of parasites circulating may strongly impair the probability of detection by PCR, while in a real transmission scenario, high densities of mosquitoes might increase the chance of infection and maintain the endemicity. Thus, these data suggest that mass treatment might be a valuable tool for disease control, although field studies are still needed to test its efficacy compared to other control measures currently employed [48].

In conclusion, we confirmed a significant proportion of asymptomatic infections in a low transmission area from the Brazilian Amazon. The distribution of asymptomatic cases in different areas is heterogeneous and is associated with a larger number of symptomatic cases in areas near the riparian forest. Low oscillating parasitemia can give false-negative results in epidemiological inquiries and the persisting silent infection fosters the conditions to turn ASY as potential reservoirs for *P*. *vivax*. Finally, using an artificial feeding assay, we show that blood from ASY infects colony-raised *N*. *darlingi*. Importantly, even with undetectable parasitemia by qPCR, ASY contribute with low rates of transmission to anophelines, suggesting their potential role in sustaining the *P*. *vivax* cycle in hypoendemic areas.
