# Correction: Asymptomatic Plasmodium vivax malaria in the Brazilian Amazon: Submicroscopic parasitemic blood infects Nyssorhynchus darlingi

**DOI:** 10.1371/journal.pntd.0011429

**Published:** 2023-06-16

**Authors:** The Negelected Tropical Diseases Genetics Staff

There is an update to the authorship. We recognize the invaluable participation of Dr Caroline Junqueira and her students, Guiherme Castro and Camila Barbosa, who helped us in performing the experiments and providing important insights during the beginning of the project. I apologize for the inconvenience of not including their names in the first version of the manuscript.

With the inclusion, the order of the authorship will be as follows:

Gregório Guilherme Almeida, Pedro Augusto Carvalho Costa, Maísa da Silva Araujo, Gabriela Ribeiro Gomes, Camila Raquel Rodrigues Barbosa, Alex Fiorini Carvalho, Maria Marta Figueiredo, Guilherme Castro, Dhelio Batista Pereira, Mauro Shugiro Tada, Jansen Fernandes de Medeiros, Irene da Silva Soares, Luzia Helena Carvalho, Flora Satiko Kano, Marcia Caldas de Castro, Joseph Michael Vinetz, Douglas Taylor Golenbock, Caroline Junqueira, Lis Ribeiro do Valle Antonelli, and Ricardo Tostes Gazzinelli.
