# Correction: Correction: Asymptomatic *Plasmodium vivax* malaria in the Brazilian Amazon: Submicroscopic parasitemic blood infects *Nyssorhynchus darlingi*

**DOI:** 10.1371/journal.pntd.0011623

**Published:** 2023-09-11

**Authors:** The Neglected Tropical Diseases Staff

The byline in the previous Correction published June 16, 2023, is incorrect. The correct byline is “The *PLOS Neglected Tropical Diseases* Staff.” The publisher apologizes for the error.

## References

[1] Almeida GG, Costa PAC, Araujo MdS, Gomes GR, Carvalho AF, Figueiredo MM, et al. (2021) Asymptomatic *Plasmodium vivax* malaria in the Brazilian Amazon: Submicroscopic parasitemic blood infects *Nyssorhynchus darlingi*. PLoS Negl Trop Dis 15(10): e0009077. 10.1371/journal.pntd.0009077 34714821 PMC8555776

[2] The *Neglected Tropical Diseases* Staff (2023) Correction: Asymptomatic Plasmodium vivax malaria in the Brazilian Amazon: Submicroscopic parasitemic blood infects Nyssorhynchus darlingi. PLoS Negl Trop Dis 17(6): e0011429. 10.1371/journal.pntd.0011429 37327217 PMC10275416

